# Probiotics in Poultry: Unlocking Productivity Through Microbiome Modulation and Gut Health

**DOI:** 10.3390/microorganisms13020257

**Published:** 2025-01-24

**Authors:** Muhammad Naeem, Dianna Bourassa

**Affiliations:** Department of Poultry Science, Auburn University, Auburn, AL 36849, USA

**Keywords:** disease resistance, growth, gut health, microbiome, probiotics

## Abstract

This review explores the role of probiotics in improving productivity and gut health in poultry through microbiome modulation, particularly during early life. Gut health is pivotal to poultry performance, influencing nutrient absorption, immune function, and disease resistance. Early-life interventions target the microbiome to shape long-term health and productivity. Probiotics, live microorganisms providing health benefits, improve gut health through the competitive exclusion of pathogens, immune modulation, antimicrobial compound production, and enhancing gut barrier integrity. Applying probiotics improves growth performance, feed conversion efficiency, body weight gain, and carcass quality by promoting lean muscle growth and reducing fat deposition. For laying hens, probiotics enhance egg production and quality. These benefits are linked to better nutrient utilization, a well-balanced microbiome, and reduced gastrointestinal disorders. However, the efficacy of probiotics depends on strain specificity, dosage, and administration methods. Factors like environmental conditions, storage stability, and interactions with other feed additives also influence their effectiveness. Despite these challenges, advancements in microbiome research and probiotic technologies, such as precision probiotics and synbiotics, provide promising solutions. Future research should focus on optimizing formulations, understanding host–microbiome interactions, and leveraging new technologies for targeted microbiome management.

## 1. Introduction

Sustainable poultry production is essential to meet the growing protein demands of an increasing population. The poultry industry has long recognized the critical importance of gut health, as it directly impacts nutrient uptake, feed efficiency, and overall animal performance. Genetic selection has been the primary driver of improved productivity in the poultry industry, accounting for 80–90% of the progress seen in broiler performance [1]. Rapid growth in poultry increases susceptibility to gastrointestinal issues, as it can disrupt the gut microbiota balance. This imbalance facilitates the colonization of pathogenic bacteria like *Salmonella*, leading to higher infection rates and associated health risks in chickens [2]. To address these challenges, researchers have explored alternative nutritional strategies, which can help to modulate the gut microbiome and improve overall gut health [3]. One promising approach to addressing this challenge is the use of probiotics, which can help to modulate the gut microbiome and improve overall gut health [1].

The gut microbiome has emerged as a cornerstone of health and productivity in poultry. This microbial ecosystem not only influences digestion and metabolism but also impacts immunity and disease resistance. In commercial poultry production, where the aim is to maximize growth and efficiency, the strategic manipulation of the gut microbiome has garnered considerable attention. The early-life manipulation of the gut microbiome using probiotics can have a lasting impact on the development and composition of the gut microbial community, with implications for long-term animal health and productivity. Numerous studies have demonstrated the potential of probiotics to improve gut health, reduce the prevalence of pathogenic bacteria, and enhance performance in poultry [3,4].

Probiotics, live microorganisms that confer health benefits to the host, offer a promising avenue for modulating the gut microbiome, particularly during the critical early-life window [5]. They, when administered in adequate amounts, confer a health benefit on the host, and they have emerged as a promising approach to enhancing gut health in poultry [3]. These beneficial bacteria can help exclude pathogens, strengthen the gut barrier, and modulate the immune response, leading to improved animal performance and reduced disease susceptibility [1]. As the poultry industry continues to seek alternatives to antibiotic growth promoters, the use of probiotics and other gut health-enhancing supplements has become an increasingly important component of sustainable and efficient poultry production [6,7,8]. This review aims to provide a comprehensive analysis of probiotics’ impacts on the poultry microbiome, highlighting their potential to enhance poultry growth by optimizing gut health and overall well-being.

## 2. Gut Microbiome and Its Role

The poultry gut microbiome plays a crucial role in feed digestion, enhances animal health by interacting with the host against pathogens, and contributes to production efficiency. Modulating its diversity and abundance can improve growth conditions and overall productivity [9]. Understanding the functions of this microbiome can lead to improved poultry management practices and enhanced production efficiency. The gut microbiota of chickens is diverse, with significant variations observed among different breeds and environmental conditions [10]. Dominant genera include *Phocaeicola* and *Bacteroides*, which are essential for metabolic processes [11]. Native chickens exhibit richer microbial diversity compared to more productive hybrid breeds, which can enhance health and production quality [11].

The gut microbiome is integral to carbohydrate metabolism, with a high prevalence of metabolic genes related to this function [11]. Short-chain fatty acids (SCFAs), particularly butyrate, produced by gut bacteria, play a vital role in regulating metabolism and immune responses [12]. The gut microbiota influences both local and systemic immunity, enhancing the host’s ability to respond to pathogens [13]. Probiotics derived from chicken gut microbiota, such as *Lactobacillus* species, have shown potential in improving immune function and alleviating intestinal disorders [11]. While the gut microbiome is essential for poultry health and productivity, it is also influenced by external factors such as diet and the environment. Future research should focus on elucidating the causal relationships between gut microbiota and host physiology to optimize poultry health management strategies.

The microbiome contributes to the following: (a) nutrient metabolism: fermentation of indigestible carbohydrates, synthesis of short-chain fatty acids (SCFAs), and vitamin production. Microbial enzymes help in breaking down complex carbohydrates and proteins, providing essential nutrients to the host. (b) Immune modulation: training the host immune system to distinguish between commensal and pathogenic microbes. A well-balanced microbiome reduces inflammation by modulating immune responses. (c) Disease resistance: competitive exclusion of pathogens through mechanisms like niche occupation and production of antimicrobial compounds. Beneficial microbes produce bacteriocins and other metabolites that inhibit pathogenic growth.

The poultry gut microbiome comprises diverse bacterial populations, primarily within the phyla *Firmicutes*, *Bacteroidetes*, *Actinobacteria*, and *Proteobacteria*. The composition evolves dynamically during the bird’s life, with the early stages being particularly influential. Initial colonization is affected by factors such as the hatching environment, maternal microbiota, and feed composition. Environmental exposure, such as contact with litter, other birds, and feed, further shapes the microbiome, with a gradual increase in microbial diversity observed over time.

## 3. Probiotics and Their Role in Gut Health

Commonly used probiotics in poultry production are given in Table 1.

These probiotic strains have been extensively studied for their ability to improve gut health, enhance growth performance, and reduce the prevalence of pathogenic bacteria in poultry.

## 4. Mode of Action and Factors Affecting the Efficacy of Probiotics

**Figure 1:**
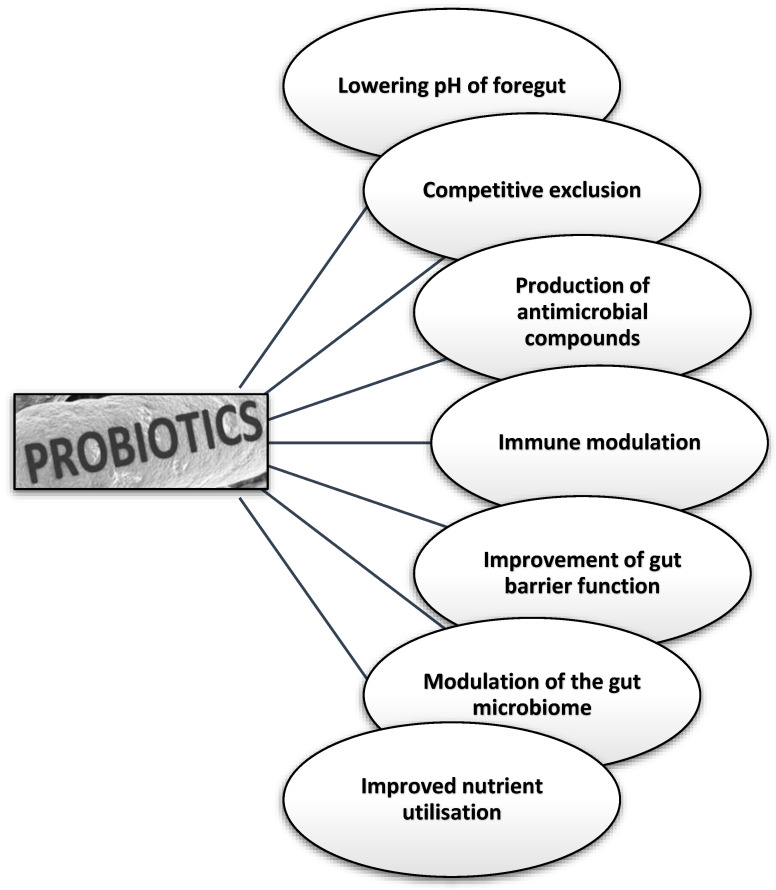
Possible modes of action of probiotics for overall improvement in poultry.

The mechanisms of action can contribute to the overall health and productivity of poultry, making probiotics a valuable tool in modern poultry production [3,14,21,22]. Probiotics exert their effects following possible modes of action given in Figure 1, while their description is given in Table 2.

The efficacy of probiotics in poultry production may be influenced by several factors, including the following: (i) probiotic strain and dose: the choice of probiotic strain and the appropriate dosage is crucial for achieving the desired effects. Different probiotic strains may have varying mechanisms of action and efficacy in poultry [27]. (ii) Timing of administration: the timing of probiotic administration can impact their effectiveness. Early-life supplementation of probiotics has been shown to have a more significant and lasting effect on the gut microbiome and animal health [28]. (iii) Diet and environmental conditions: the composition of the feed, as well as the environmental factors, such as housing, temperature, and biosecurity measures, can affect the survival and colonization of probiotics in the gut [29]. (iv) Host factors: the age, genetics, and immune status of the poultry can also influence the response to probiotic supplementation [30]. (v) Interaction with other feed additives: probiotics may interact with other feed additives, such as antibiotics, enzymes, and prebiotic fibers, which can either enhance or diminish their efficacy [31]. Understanding and optimizing these factors can help maximize the benefits of probiotic supplementation in poultry production.

## 5. Early-Life Gut Microbiome and Factors Affecting Its Development

Early colonization has long-term consequences on gut health and overall productivity. Proper microbial establishment influences metabolic programming, immune system development, and resistance to environmental stressors. Inadequate colonization during this period can result in dysbiosis, increasing susceptibility to diseases and reducing growth efficiency. Early-life manipulation of the gut microbiome using probiotics can have a lasting impact on the development and composition of the gut microbial community, with implications for long-term animal health and productivity. Numerous studies have demonstrated the potential of probiotics to improve gut health, reduce the prevalence of pathogenic bacteria, and enhance performance in poultry [3,4].

Key factors influencing early microbiome development may include the following: (i) hatchery practices: hygiene and microbial exposure. Contaminated hatcheries can introduce harmful microbes, whereas probiotic sprays at hatching can promote beneficial colonization. (ii) Maternal microbiota: vertical transmission of beneficial microbes. The maternal microbiome impacts egg microbiota and chick gut colonization. (iii) Diet: early feeding strategies, including prebiotics and probiotics. First feedings establish gut microbial diversity and functionality. Delayed access to feed post-hatch can hinder proper microbial development

## 6. Impact of Probiotics on Early-Life Microbiome Development

Administering probiotics during early life can prevent opportunistic pathogens from dominating the microbiome contributing to a rapid establishment of beneficial microbes. A chick usually develops its gut microbiota through exposure to the feces of adult chickens. However, in a hatchery setting, where contact with mature chickens is absent, probiotic supplementation proves advantageous [6]. Probiotic-fed chicks exhibit higher levels of mucin production and tighter gut epithelial junctions contributing to enhanced gut barrier function and immunity [22]. Probiotics can reduce stress-associated cortisol levels and help maintain microbial equilibrium from the early life of chicks [24], mitigating stress-induced dysbiosis during critical periods such as transportation and housing.

Studies have shown that the early-life administration of probiotics can have a lasting impact on the composition and diversity of the gut microbiome in poultry. For example, one study found that the supplementation of broiler diets with a multi-strain probiotic during the first week of life resulted in significant changes in the gut microbiome, including an increase in the abundance of beneficial bacteria, such as *Lactobacillus* and *Bifidobacterium*, and a reduction in the prevalence of pathogenic bacteria, such as *Escherichia coli* and *Clostridium* [32]. By administering probiotics at hatch time, Baldwin et al. [33] also found that *Lactobacillus* strains significantly influenced gut microbiota development, promoting beneficial taxa and reducing pathogenic ones, ultimately enhancing weight gain and improving overall gut health and performance in broiler chickens. These findings suggest that the early-life manipulation of the gut microbiome through probiotics can have long-lasting benefits for poultry health and production [34,35,36].

## 7. Impact of Probiotics on Gastrointestinal Tract Microbiome

The crop is an important part of the gastrointestinal tract in poultry, serving as a temporary storage and fermentation chamber for ingested feed. The composition of the crop microbiome can have a significant impact on the overall health and performance of poultry. Studies have shown that the supplementation of poultry diets with probiotics can modulate the crop microbiome, leading to a reduction in the prevalence of pathogenic bacteria and an increase in the abundance of beneficial bacteria. For instance, previous research reported that the inclusion of a *Bacillus*-based probiotic in the diet of broiler chickens led to a significant reduction in the abundance of *Escherichia coli* and *Salmonella* in the crop while increasing the levels of *Lactobacillus* and *Bifidobacterium* species [37]. Even in layers, it has been reported that the supplementation of laying hens’ diets with a probiotic containing *Lactobacillus* and *Bifidobacterium* species resulted in a more diverse and balanced crop microbiome, with a reduction in the prevalence of pathogenic bacteria [38].

The gut microbiome plays a crucial role in the overall health and performance of poultry. The effect of probiotics on the intestinal microbiome in poultry is significant, as they enhance gut health, improve growth performance, and serve as alternatives to antibiotics. *Enterococcus* faecium and *Bacillus* subtilis probiotics modulate the intestinal microbiota, promoting beneficial bacteria and reducing pathogenic strains, which is crucial for poultry health and productivity.

Since probiotics increase the abundance of beneficial bacteria, which are essential for gut health, research has shown that the supplementation of compound probiotics significantly enhanced the abundance of beneficial bacteria such as *Lactobacillus* and *Faecalibacterium*, modulated cecal microbiota structure, and promoted the production of SCFAs, thereby improving intestinal health in poultry [39]. Probiotics help maintain a balanced microbiome, combating opportunistic pathogens and enhancing overall gut function [40]. Despite the inconsistencies in the outcome response of probiotics, various studies have indicated that probiotics positively influence the intestinal microbiome, enhancing productivity and overall health in poultry.

Under stress conditions, probiotics can modulate the gut microbiota in poultry by promoting beneficial bacterial colonization, enhancing intestinal barrier integrity, and influencing immune pathways. These interactions contribute to improved gut health and overall bird productivity, particularly under stress conditions [41]. Some studies have reported a reduced environmental footprint with the use of probiotics as they significantly improved the intestinal microflora in broiler chickens by increasing lactic acid bacteria and enhancing the richness and variety of microflora compared to antibiotics and control groups, while also reducing fecal emissions of ammonia and sulfured hydrogen [42].

In the ileum section, probiotics significantly modulated broiler gut microbiota, with viable probiotic forms increasing ileal *Bacteroides* spp. and cecal *Lactobacillus* spp. levels, while inactivated forms showed lesser effects, highlighting the importance of probiotic viability for beneficial gut microbiome modulation in poultry [43]. Grozina et al. [44] reported that the *Bacillus* subtilis strain positively influenced the microbiological composition of the gastrointestinal tract in broiler chickens, enhancing growth and regulating intestinal microflora, thereby improving feeding efficiency and overall productivity. The administration of synbiotics (probiotics and prebiotics) in turkeys significantly increased beneficial microorganisms (*Lactobacilli* and *Bifidobacterium* spp.), while reducing potential pathogens (*Clostridium* spp. and *Escherichia coli*), demonstrating a positive effect on the intestinal microbiome in poultry [45].

Conversely, skepticism exists regarding the effectiveness of probiotics in poultry, with some studies indicating limited impact on microbiome modulation and growth performance, suggesting that more research is needed to fully understand their benefits [40]. However, this variability in the efficacy may be due to differences in the strains of probiotics used, the age of the birds, or other methodologies of inclusion, which warrant further investigations.

The cecum is another important part of the poultry gastrointestinal tract, serving as a site for the fermentation of complex carbohydrates and the production of volatile fatty acids. The composition of the cecal microbiome can have a significant impact on the overall health and performance of poultry. Overall, supplementing poultry diets with probiotics can modulate the cecal microbiome, leading to a reduction in the prevalence of pathogenic bacteria and an increase in the abundance of beneficial bacteria [7]. Hoepers et al. [46] observed that probiotics significantly altered the cecal microbiome composition in broilers, as indicated by metagenomic analysis. Supplementation led to changes in predominant bacterial families and genera, enhancing gut health and potentially reducing oxidative stress and intestinal inflammation.

The inclusion of a probiotic containing *Bacillus*, *Lactobacillus*, and *Bifidobacterium* species in the diet of broiler chickens resulted in a significant increase in the abundance of butyrate-producing bacteria in the caecum, which can have a positive impact on gut health and overall animal performance [8]. However, probiotics, like *Bacillus* amyloliquefaciens H57, significantly alter the functional capacity of the poultry cecal microbiome, enhancing amino acid and vitamin biosynthesis pathways without significantly affecting microbial community composition, thus improving overall chicken productivity [47]. The species *Bacillus* pumilus and *Bacillus* subtilis significantly enhance the early maturation of cecal microbiota in broiler chickens, increasing beneficial bacteria like *Lactobacillus* and *Bifidobacterium* while decreasing harmful *Enterobacteriaceae*, thus promoting healthier gut microbiota configurations [48].

It appears that most of the probiotics, which altered cecal microbiota profiles, enhanced beneficial *Lactobacillales*, and reduced harmful *Clostridia* in poultry, were correlated with improved body weight and lower mortality rates, demonstrating their potential to modulate intestinal microbiota effectively [49]. A reduction in *Enterobacteriaceae* in the ceca and reversing the effects of *Salmonella* infection, enhancing *Firmicutes* levels, and improving overall gut health and microbial diversity in broiler chickens have also been witnessed [50]. The increase in the abundance of *Enterobacteriaceae* in the ceca and feces of chickens was correlated with enhanced IgA production against *E. coli* virulence factors. This indicates that probiotics positively influence the gut microbiome composition in poultry [51,52]. The microbiota of chickens with an abundance of beneficial bacteria like *Prevotella* and *Bacteroides* enhance microbial diversity and promote the production of short-chain fatty acids, which are crucial for gut health and nutrient utilization [53].

It seems single-strain probiotics modulate cecal microbiota in broilers with low diversity [54]. On the other hand, Chang et al. [55] found that multi-strain probiotics significantly increased *Lactobacillus* populations in broiler ceca, improved intestinal microbiota composition, and decreased harmful bacteria like *Escherichia* spp. and *Clostridium* perfringens, enhancing overall gut health and microbiome balance in poultry. In laying hens, supplementing diets with *Lactobacillus* and *Bifidobacterium* species led to an increase in the diversity of the cecal microbiome, with a reduction in the prevalence of *Salmonella* and *Clostridium* species [6].

## 8. Impact of Probiotics on Gut Health

The significant improvement in the gut microbiota of broilers is caused by increasing beneficial microbes like *Bacteroides* and decreasing harmful ones such as *Proteobacteria*, enhancing intestinal health and overall growth performance [56]. *Bacillus* subtilis probiotic supplementation has been observed to enrich gut microbiota in poultry, specifically increasing *Streptococcus*, *Butyricicoccus*, *Faecalibacterium*, and *Ruminococcus*. This enrichment upregulated genes related to intestinal health, enhancing the physical barrier and reducing inflammation, thus promoting overall gut function [57]. Ferrocino et al. [58] explained that *Lactiplantibacillus* plantarum and *Lactiplantibacillus* pentosus increased the abundance of beneficial bacteria like *Blautia* and *Faecalibacterium* in broilers, enhancing gut microbiota composition and function and promoting a healthier gut status without impairing overall health or performance. Similarly, the introduction of *Bacillus* megaterium and *Enterococcus* faecium strains increased *Bacillaceae* by 36% and *Veillonellaceae* by 60%, while reducing pathogenic species like Enterobacteriaceae by 1.25 times, positively impacting the poultry microbiome [59]. *Bacillus* coagulans and *Lactobacillus* plantarum increase the abundance of *Ruminococcaceae* and reduce *Desulfovibrio* in broilers, enhancing intestinal microbiota diversity and promoting overall gut health [60].

*Lactobacillus* plantarum and *Bifidobacterium* longum enhance gut health in poultry by improving gastrointestinal microbial balance, inhibiting pathogenic microbes and promoting beneficial interactions with the intestinal mucosa, ultimately supporting better feed utilization and overall poultry performance [61]. *Bacillus* subtilis are reported to positively influence poultry microbiomes by increasing the abundance of beneficial genera like *Lactobacillus*, enhancing butyrate production, and modulating anti-inflammatory functions, thereby improving gut homeostasis and overall health during enteric infections [62]. Mazanko et al. [63] observed that *Bacillus* probiotics, particularly KB41 and KB54, colonized the intestines of broilers, enhancing growth performance and immune modulation, indicating their positive influence on the poultry microbiome and overall health. Additionally, Khan and Chousalkar [64] reported that probiotic supplementation in poultry, particularly *Bacillus*-based probiotics, restores gut microbial balance disrupted by *Salmonella* Typhimurium infection, enhancing microbial diversity, increasing butyrate and propionate levels, and significantly reducing *Salmonella* load in feces and internal organs. *Lactobacillus* casei modulates the poultry gut microbiome by reducing *Proteobacteria* and increasing *Firmicutes*, enhancing bacterial diversity, and decreasing the colonization of harmful pathogens like *Campylobacter* jejuni and *Salmonella* enterica, promoting a healthier gut environment [65]. Significant negative correlations observed between known probiotics (*Bacillus*) and *Campylobacter* in poultry microbiomes also suggest that probiotics may help reduce pathogen prevalence [66].

Even in disease-challenged conditions like necrotic enteritis, probiotics enhance the gut microbiome in poultry by promoting beneficial microbial colonization, inhibiting pathogenic bacteria, and modulating immune responses, which leads to improved gut health, nutrient absorption, and overall growth performance [67]. Both postbiotics and paraprobiotics positively affect the poultry microbiome by increasing beneficial microbes like *Firmicutes* and decreasing harmful microbes such as *Proteobacteria*, leading to a healthier gut microbiota in broiler chickens [68]. Whereas, in the hindgut, probiotics increase the relative abundance of beneficial *Lactobacillus* in the ileal mucosa of broiler chickens while reducing harmful taxa such as *Clostridium* sensu stricto 1 and *Ruminiclostridium* 9, thereby positively influencing gut health and microbiome balance [25].

Not only in broilers but also in laying hens do *Bacillus*-based probiotics increase *Bacteroidetes* and *Proteobacteria* while decreasing *Firmicutes*, enhancing gut health. They also enrich metabolic pathways, improve egg internal quality, and reduce *Salmonella* isolation, indicating positive effects on the gut microbiome [69]. Xu et al. [70] reported that probiotics significantly altered the gut microbiota of laying hens, increasing the distribution of *Firmicutes*, *Bacteroidota*, and *Synergistota* during early laying, while enhancing *Bacteroidota*, *Actinobacteriota*, and *Verrucomicrobiota* at peak production, and *Firmicutes* and *Desulfobacterota* in late laying periods. In ducks, it is also observed that probiotics significantly alter the microbiome by decreasing the opportunistic pathogens *Escherichia* and *Shigella* and enhancing the diversity of beneficial bacterial species, leading to improved health and production performance in ducks fed with *Bacillus*-probiotic-enriched feed [71].

The literature suggests that probiotics in poultry primarily enhance the microbiome by promoting beneficial microbial strains, which outcompete harmful microorganisms, improve immunological responses, and increase the overall health and productivity of the birds, thereby reducing the reliance on antibiotics in poultry production [72]. Secondly, probiotics maintain normal intestinal microflora in poultry through competitive exclusion and antagonistic interactions, enhancing digestive enzyme activity while reducing harmful bacterial activity and ammonia production [73]. Thirdly, probiotics can positively alter the gut microbiota in poultry by preventing dysbiosis, enhancing microbial diversity, and improving growth, performance, and immune parameters. This may be achieved through mechanisms like competition for resources and secretion of antimicrobial substances against pathogenic microbes [74].

## 9. Impact of Probiotics on Gut Morphology

Supplementing probiotics positively affects gut morphology by increasing villus height and crypt depth, enhancing nutrient absorption capacity [22]. Improved epithelial integrity reduces the risk of bacterial translocation, as probiotics increase the length of intestinal villi and reduce crypt depth [75]. Longer villi enhance nutrient absorption by providing a larger surface area. Crypts are the sites of enterocyte proliferation, and shallower crypts suggest a reduced need for epithelial cell turnover, allowing for energy typically used for cell renewal to be redirected towards growth [76]. Another example of probiotics improving gut health by improving intestinal histomorphology was observed when mycotoxin deoxynivalenol was added to feed [77]. While mycotoxin deoxynivalenol typically damages the gut by reducing villus height and width in the duodenum and jejunum, probiotic treatment mitigated these harmful effects [77]. Mehdi et al. [4] reported that the use of a probiotic containing *Bacillus* subtilis in broiler breeder diets improved intestinal morphology, enhanced nutrient absorption, and reduced the incidence of necrotic enteritis. It is observed that probiotic *Lactobacilli* treatment at a dose of 1 × 10^8^ CFU significantly improves gut morphology in poultry by increasing villus height in the ileum and reducing crypt depth in the jejunum. These changes contribute to enhanced intestinal health and function, which are crucial for mitigating necrotic enteritis caused by *Clostridium* perfringens [78]. This highlights the potential of probiotics as a beneficial alternative to antibiotics in poultry management, promoting better gut structure and immune responses [78]. Similarly, when duo-strain probiotics were supplemented, they significantly enhanced the tissue morphology of the duodenum and jejunum in broiler chickens after four weeks [79]. Specifically, the duo-strain probiotics treatment increased microvillus height in these intestinal regions, which is crucial for nutrient absorption and provides an enlarged surface area of the villi. However, there were no notable effects observed in the ileum with the supplementation of due-strain probiotics [79]. In brief, this suggests that the inclusion of probiotics in poultry diets can positively influence gut morphology, potentially leading to improved digestive efficiency and overall gut health.

## 10. Impact of Probiotics on Disease Resistance

Probiotics have a significant impact on disease resistance in poultry by enhancing the immune system and safeguarding gut microflora. These live, non-pathogenic microorganisms improve the overall health and well-being of the host, leading to better production performance and immunity [27]. Probiotics, such as the preparation of Immunoflor, significantly enhance disease resistance in poultry by strengthening humoral factors of natural resistance. In a study, broiler chickens receiving Immunoflor showed increased lysozyme and bactericidal activity in blood serum by 10.5–17.2% and 8.5–12.1%, respectively. Additionally, the phagocytic properties of blood leukocytes improved, and there was an increase in the mass of immunocompetent organs, leading to better overall health and population safety in the experimental groups [80]. The efficacy of probiotics is well demonstrated against common poultry pathogens, including *Salmonella* spp., as they reduce colonization of pathogens in the gut. Reduced colonization of *Clostridium* perfringens and *Escherichia coli* by supplementing probiotics has been observed. This leads to improved growth performance, feed utilization, disease resistance, and overall health, demonstrating their significant positive impact on the poultry microbiome [81]. For instance, *Bacillus* subtilis has shown efficacy in preventing necrotic enteritis and reducing *Salmonella* colonization, thereby improving intestinal health and food safety [46,57]. Similarly, *Lactobacillus* species enhance the intestinal microbial balance in poultry, improving resistance to pathogens like *Escherichia coli* and *Salmonella* spp., while restoring normal flora, aiding digestion, and boosting the immune system against infectious diseases [82], thereby contributing to overall health and disease resistance in chickens, especially during early life stages [83]. A complex of *Bifidobacteria* and *Lactobacilli* also enhances colonization resistance against pathogenic microflora [84]. Probiotics exert antagonistic effects on various microorganisms in poultry by improving gut epithelial barrier function, competing for adhesive receptors and nutrients, and providing antibacterial effects. This modulation of the microbiome appears to contribute to enhanced health and growth performance in poultry.

## 11. Impact of Probiotics on Immune Modulation

Probiotics play a significant role in immune modulation, influencing various immune cells and pathways that contribute to health and disease management. Their effects are particularly notable in enhancing the immune response, regulating inflammation, and promoting gut health, which collectively support the body’s defence mechanisms. The impact of probiotics on immune modulation in poultry is significant, as they enhance the immune response and overall health of birds. For instance, *Bacillus* strains and Immunoflor have been shown to improve immune function by modulating cytokine expression and enhancing natural resistance factors [80]. These probiotics, e.g., Immunoflor, also enhance immune modulation in poultry by increasing humoral resistance factors, lysozyme and bactericidal activity, and improving phagocytic properties of leukocytes, ultimately leading to better population safety and health in broiler chickens [80]. This modulation is crucial for reducing reliance on antibiotics in poultry farming. Probiotics play a role in cytokine regulation. Probiotics like *Bacillus* subtilis and *Bacillus* velezensis upregulate anti-inflammatory cytokines (e.g., IL-10) and immune cell markers while downregulating pro-inflammatory cytokines [85]. Probiotics also act as natural resistance enhancers. The probiotic Immunoflor increases lysozyme and bactericidal activity in broiler chickens, improving their humoral immune response [80].

Innate and adaptive immunity through probiotics enhance the production of secretory IgA and cytokines, thereby improving disease resistance. Research has demonstrated increased macrophage activity and lymphocyte proliferation in probiotic-supplemented birds. Khan et al. [86] observed that *Lactobacillus* and *Bifidobacterium* species enhance the gut microbiome in poultry by promoting beneficial microbial populations, producing antimicrobial agents, and activating the host immune system, which collectively improves gut health and resistance to *Salmonella* and *Campylobacter*. Furthermore, probiotics enhance poultry microbiome by restoring gut microbial balance, increasing beneficial bacteria like *Lactobacillus* and *Bacillus*, and reducing pathogenic *Clostridium* perfringens. They improve mucosal integrity, modulate immune responses, and promote overall gut health, thus preventing diseases like necrotic enteritis [87].

## 12. Impacts of Probiotics on the Growth Performance of Poultry

Supplementing poultry diets with probiotics can lead to improvements in feed conversion ratio, body weight gain, and overall animal productivity, which may be due to the enhanced digestibility of nutrients [88], modulating gut microbiome with increased beneficial and reduced pathogenic microbiota, or strengthening the gut barrier [3,39,44]. Improvements in growth have been observed through enhanced poultry microbiome by increasing beneficial *Bifidobacteria* and *Lactobacilli* while reducing harmful *Staphylococci* and *Enterococci*, optimizing intestinal microbiota composition and improving feed digestibility and body weight dynamics in broiler chickens [89]. Probiotics also promote the synthesis of antimicrobial peptides, which can lead to better nutrient utilization, growth performance, and overall immunity, contributing to healthier poultry production compared to antibiotic use [90]. An outcompeting mechanism for harmful microorganisms is obvious from the research that probiotics follow to improve growth performance as they promote beneficial microbial strains, which can outcompete harmful microorganisms, produce antibacterial compounds, and modulate the host’s immune response, ultimately improving overall health, growth, and feed efficiency in chickens [27,30,72]. Probiotics also enhance digestive enzyme activity, leading to better nutrient absorption and improved growth performance [39].

Many studies have demonstrated that the use of probiotics in poultry diets can positively impact feed consumption and feed conversion efficiency. Mehdi et al. [4] reported enhanced nutrient absorption with the use of a probiotic containing *Bacillus* subtilis in broiler breeder diets. However, using multi-strain probiotics, Korver [88] reported an improved feed conversion ratio, increased weight gain, and reduced *Salmonella* colonization compared to control birds. Similarly, Saili et al. [91] found that supplementing diets with multi-strain probiotics resulted in a significant improvement in feed intake and feed conversion ratio, leading to enhanced growth performance. These researchers reported that the supplementation of laying hens’ diets with a probiotic containing *Bacillus* subtilis and *Enterococcus* faecium led to increased feed intake and better egg production [91]. This improved feed consumption and feed conversion efficiency observed with probiotic supplementation may be attributed to enhanced nutrient digestibility and absorption due to the modulation of the gut microbiome, improved gut health and reduced incidence of gastrointestinal disorders, increased appetite and feed palatability, reduced stress, and improved overall animal welfare [3]. These benefits can contribute to more efficient feed utilization and ultimately improved productivity and profitability in poultry production [4,91].

The improved feed conversion ratio associated with probiotic supplementation can have a significant impact on the overall profitability of poultry production, as it reduces the amount of feed required to achieve a certain level of growth or egg production. Studies have consistently shown that the use of probiotics in poultry diets can lead to a significant improvement in the feed conversion ratio, which is a measure of the efficiency of feed utilization [3,91]. The supplementation of broiler diets with a probiotic containing *Lactobacillus*, *Bifidobacterium*, and *Enterococcus* species resulted in a significant reduction in the feed conversion ratio compared to the control group [92]. Another study using *Bacillus* amyloliquefaciens reported a reduction of 0.08 kg/kg in FCR [93]. A meta-analysis of 37 studies on the use of probiotics in broiler chickens found that probiotic supplementation led to an average reduction of 0.06 in the feed conversion ratio, indicating a more efficient feed utilization [73].

**Figure 2:**
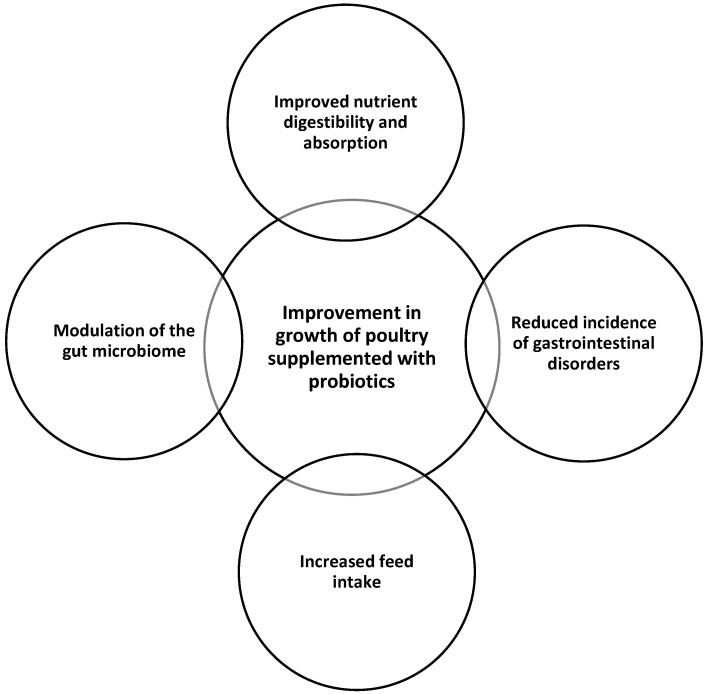
Possible mechanisms of probiotics in improving growth in poultry.

Briefly, probiotic supplementation at recommended doses has been associated with improved performance metrics, including FCR, carcass yield, and bird welfare [94]. The improvements in growth rate and feed conversion ratio observed with probiotic supplementation may be attributed to the following: (i) improved nutrient digestibility and absorption: probiotics can enhance the production of digestive enzymes, increase the surface area of the intestine, and promote the absorption of nutrients, leading to more efficient feed utilization. (ii) Reduced incidence of gastrointestinal disorders: probiotics can inhibit the growth of pathogenic bacteria, strengthen the gut barrier, and modulate the immune system, thereby reducing the occurrence of digestive issues that can impair feed efficiency. (iii) Increased feed intake: probiotics have been shown to stimulate appetite and feed intake, which can contribute to better growth and feed conversion. (iv) Modulation of the gut microbiome: probiotics can favorably alter the composition and diversity of the gut microbiome, creating a more favorable environment for nutrient utilization and overall animal health. Possible mechanisms of probiotics in improving growth in poultry are given in Figure 2.

## 13. Impact of Probiotics on Carcass

The impact of probiotics on carcass quality in poultry has garnered significant attention due to their potential as alternatives to antibiotics. The use of probiotics in poultry production has also been shown to have a positive impact on carcass characteristics, such as meat yield, fat deposition, and meat quality. The improvement in the carcass may be due to muscle development as the administration of probiotics during specific growth phases resulted in improved muscle tissue development, leading to better overall growth metrics [84]. Probiotic-fed broiler chicks exhibited improved carcass characteristics, including better meat quality with higher protein and fiber content and lower fat, ash, and nitrogen-free extract levels, indicating that probiotics positively influence carcass traits compared to antibiotic treatments [95]. Previous research [96] found that the dietary supplementation of broiler chickens with a probiotic containing *Lactobacillus*, *Bifidobacterium*, and *Enterococcus* species resulted in a significant increase in breast meat yield compared to the control group without probiotics. The inclusion of a *Bacillus*-based probiotic in the diet of broiler chickens also led to a reduction in abdominal fat deposition and an improvement in the fatty acid composition of the meat, with higher levels of unsaturated fatty acids [97]. The improved carcass characteristics associated with probiotic supplementation can have a significant impact on the profitability of poultry production, as it can lead to increased meat yield, improved meat quality, and reduced feed costs.

**Figure 3:**
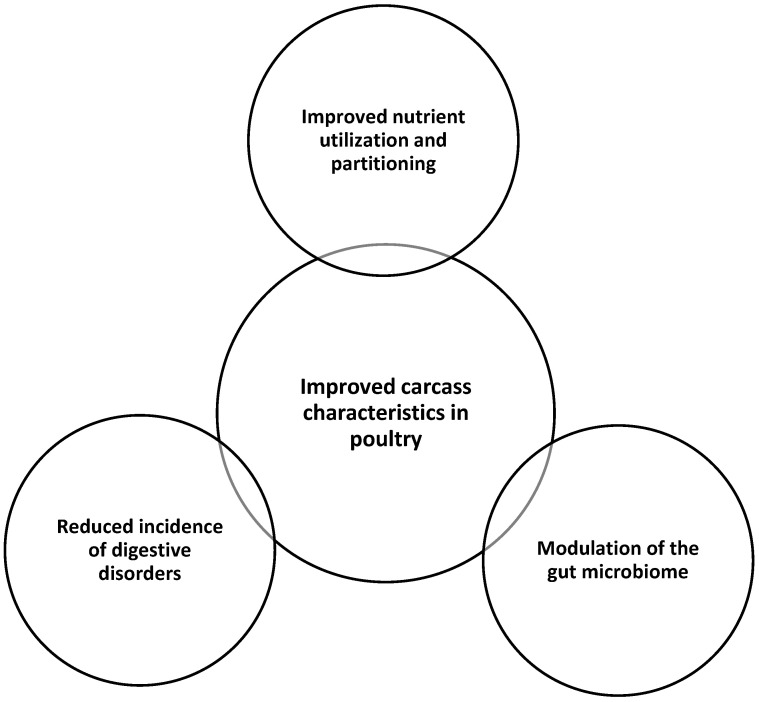
Possible mechanisms of probiotics in improving carcass characteristics in poultry.

The mechanisms by which probiotics can improve carcass characteristics in poultry may include the following: (i) improved nutrient utilization and partitioning: probiotics can enhance the absorption and utilization of nutrients, such as protein and energy, leading to increased muscle growth and reduced fat deposition. (ii) Modulation of the gut microbiome: probiotics can favorably alter the composition and diversity of the gut microbiome, creating a more optimal environment for nutrient utilization and overall animal health. (iii) Reduced incidence of digestive disorders: probiotics can inhibit the growth of pathogenic bacteria, strengthen the gut barrier, and modulate the immune system, reducing the occurrence of digestive issues that can impair growth and carcass quality. Possible mechanisms of probiotics in improving carcass characteristics in poultry are given in Figure 3.

## 14. Impact of Probiotics on Egg Production in Laying Hens

The use of probiotics in the diets of laying hens has been shown to have a positive impact on egg production and quality. Supplementation of laying hen diets with various probiotic strains, such as *Lactobacillus*, *Bifidobacterium*, and *Bacillus*, can lead to an increase in egg production and improved egg quality. Probiotic supplementation, particularly with *Bacillus* and *Lactobacillus* strains, has been shown to increase egg production rates. For instance, hens receiving 0.2% *Lactobacillus* spp. exhibited higher egg production in specific weeks compared to control groups [98]. In another study, hens supplemented with Bacillus-based probiotics demonstrated improved hen-day egg production and average egg weight, particularly at higher dosages [99]. However, *Lactobacillus* rhamnosus significantly improves egg production performance in late-phase laying hens by decreasing the feed conversion ratio and enhancing eggshell quality, ultimately leading to better overall production outcomes compared to control groups [100].

Tsai et al. [101] observed that the inclusion of a *Bacillus*-based probiotic in the diet of laying hens resulted in a 5% increase in egg production, as well as improvements in egg weight, shell thickness, and Haugh unit, a measure of egg freshness and quality. Research by Lui et al. [100] and Abdelqader et al. [96] demonstrated that the supplementation of laying hens’ diets with probiotics containing *Lactobacillus* and *Bifidobacterium* species led to an increase in egg production, egg weight, and eggshell quality. An increase of 5% in egg production, as well as improvements in egg weight, shell thickness, and Haugh unit, has been observed through the inclusion of a *Bacillus*-based probiotic in the diet of laying hens [96]. Enhanced feed efficiency and reduced incidence of disease through probiotics result in increased egg production, improved egg quality, and increased profitability of egg production [100,102,103].

**Figure 4:**
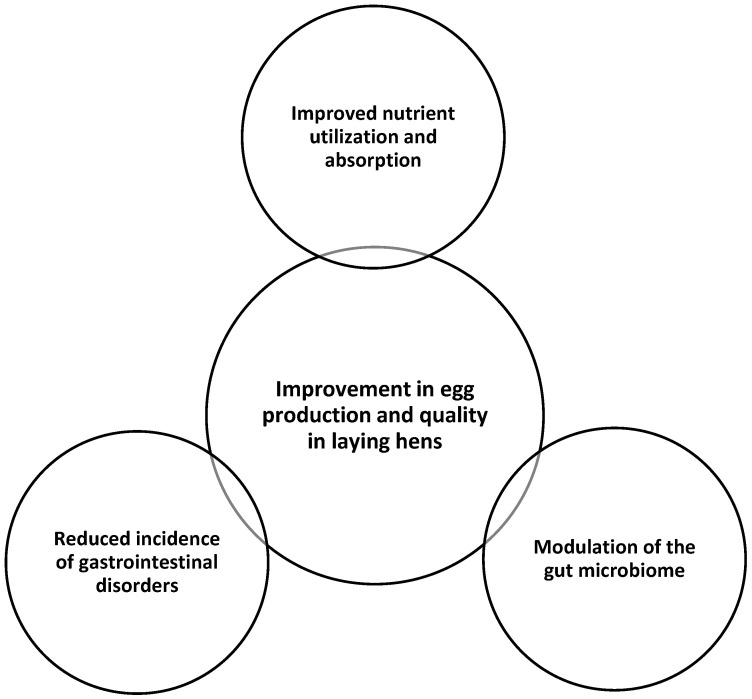
Possible mechanisms of probiotics in improving egg production and quality in laying hens.

The mechanisms by which probiotics can enhance egg production and quality in laying hens may include the following: (i) improved nutrient utilization and absorption: probiotics can enhance the production of digestive enzymes and improve the absorption of nutrients, such as calcium, which are essential for eggshell formation. (ii) Modulation of the gut microbiome: probiotics can favorably alter the composition and diversity of the gut microbiome, creating a more optimal environment for nutrient utilization and overall animal health. (iii) Reduced incidence of gastrointestinal disorders: probiotics can inhibit the growth of pathogenic bacteria, strengthen the gut barrier, and modulate the immune system, reducing the occurrence of digestive issues that can negatively impact egg production. Possible mechanisms of probiotics in improving egg production and quality in laying hens are given in Figure 4. It appears the benefits observed are likely due to the ability of probiotics to modulate the gut microbiome, improve nutrient utilization, and reduce the incidence of gastrointestinal disorders in laying hens. The improved egg production and quality associated with probiotic supplementation can have a significant impact on the profitability of egg production, as it can lead to increased yields and improved product quality.

Overall, probiotics improve the intestinal microbial balance in poultry by stimulating beneficial microorganisms and reducing pathogenic loads. This leads to enhanced gut health, lower gastrointestinal disease risks, and overall better growth performance, feed conversion, and product quality in commercial poultry production.

## 15. Challenges and Future Perspectives

Probiotics’ efficacy is influenced by factors such as strain specificity. Not all strains are equally effective in poultry [104]. Dosage and administration methods or inconsistent delivery methods can affect outcomes [105]. Stressors like temperature fluctuations and breed differences can modulate probiotic effectiveness. Ensuring the stability of probiotics in feed and during storage is a significant challenge [106]. Encapsulation and spore-forming probiotics like *Bacillus* have been developed to address this issue. Storage conditions and pelleting processes often reduce microbial viability [107]. Regulatory approval processes and the cost of production can limit widespread adoption. Variability in global regulations regarding probiotic claims adds complexity for producers.

Advancements in microbiome research may enable the development of strain-specific probiotics tailored to individual needs. Precision probiotics; a targeted approach to probiotics, which could effectively account for the variability in probiotic strains, hosts, and their microbiomes [108], could target specific pathogens or metabolic deficiencies. Combining probiotics with prebiotics (synbiotics) offers synergistic benefits by enhancing the survival and colonization of beneficial microbes [109]. Prebiotics like inulin or fructo-oligosaccharides act as substrates for probiotics, which may enhance their efficacy to yield profound synergistic effects. Emerging candidates, such as engineered probiotics and postbiotics, paraprobiotics, synbiotics, and phytobiotics, represent a new frontier in gut health management. Postbiotics, non-viable microbial cells, or their metabolites can exert similar health benefits without the viability challenges of probiotics. Real-time monitoring of the gut microbiome using omics technologies could inform probiotic interventions, aligning with precision farming principles. Integration with wearable sensors and data analytics may enable targeted microbial management.

## 16. Conclusions

The body of evidence presented in this review suggests that the inclusion of probiotics in poultry diets can have significant benefits for gut health, growth performance, and carcass quality. By modulating the composition and diversity of the gut microbiome, probiotics can improve nutrient utilization, reduce the incidence of digestive disorders, and enhance the overall health and well-being of poultry. While the potential benefits of probiotics in poultry production are well-documented, there are still gaps in our understanding of the complex interactions between the gut microbiome, host physiology, and environmental factors. Further research is needed to optimize probiotic formulations, identify the most effective delivery methods, and elucidate the mechanisms by which probiotics can modulate the gut microbiome and improve animal health. As our understanding of the poultry microbiome deepens, the integration of probiotics into holistic gut health management strategies will likely become a cornerstone of sustainable poultry production.

## Figures and Tables

**Table 1:** Commonly used probiotics in poultry production.

Probiotics	Description	References
*Lactobacillus* species	*Lactobacillus* acidophilus, *Lactobacillus* reuteri, *Lactobacillus* fermentum, and *Lactobacillus* johnsonii. They enhance gut barrier integrity and SCFA production. These strains are typically abundant in the crop and small intestine.	[3,4,14]
*Bifidobacterium* species	*Bifidobacterium* bifidum, *Bifidobacterium* longum, and *Bifidobacterium* animalis. They modulate immune responses and reduce pathogenic load. These microbes are more prevalent in the cecum.	[15]
*Bacillus* species	*Bacillus* subtilis, *Bacillus* cereus, and *Bacillus* coagulans. *Bacillus* is known for spore-forming capability, ensuring stability in feed. *Bacillus* strains are widely used due to their resilience during processing and storage of feed.	[16]
*Enterococcus* species	*Enterococcus* faecium. They contribute to gut health through antimicrobial peptide production and pathogen inhibition.	[17]
*Streptococcus* species	They contribute to gut health by modulating intestinal microbiota and producing SCFAs.	[18,19]
*Saccharomyces* cerevisiae (yeast)	They help in the fermentation process of indigestible materials in the intestinal tract. During fermentation, S. cerevisiae produces bioactive compounds such as β-glucans and prebiotic oligosaccharides, which promote gut health and enhance the immune response.	[20]

**Table 2:** Possible modes of action of probiotics for overall beneficial effects.

Mode of Action	Description	References
Lowering pH	Probiotics work by reducing gut pH, which is achieved through the production of volatile fatty acids and organic acids during the breakdown of the probiotic product. Low pH conditions inhibit pathogenic organisms’ growth, especially in the foregut.	[22]
Competitive exclusion	Probiotics can compete with pathogenic bacteria for nutrients, attachment sites, and other resources within the gut, thereby preventing their colonization and proliferation.	[23]
Production of antimicrobial compounds	Certain probiotic strains can produce a variety of antimicrobial compounds, such as organic acids, hydrogen peroxide, and bacteriocins, which can inhibit the growth of harmful bacteria.	[24]
Immune modulation	Probiotics can stimulate the host’s immune system, enhancing the production of antimicrobial compounds, increasing the activity of immune cells, and promoting a robust immune response against pathogens.	[24]
Improvement of gut barrier function	Probiotics can strengthen the gut barrier, reducing intestinal permeability and preventing the translocation of pathogenic bacteria.	[25]
Modulation of the gut microbiome	Probiotics can influence the composition and diversity of the gut microbial community, promoting the growth of beneficial bacteria and excluding the colonization of harmful microorganisms.	[9]
Improved nutrient utilization	Probiotics can improve the digestion and absorption of nutrients, leading to more efficient feed conversion and better growth performance.	[14,26]

## Data Availability

Not applicable.
